# A Comparative Biology of Microglia Across Species

**DOI:** 10.3389/fcell.2021.652748

**Published:** 2021-04-01

**Authors:** Kaushik Sharma, Kanchan Bisht, Ukpong B. Eyo

**Affiliations:** ^1^Center for Brain Immunology and Glia, University of Virginia, Charlottesville, VA, United States; ^2^Department of Neuroscience, University of Virginia, Charlottesville, VA, United States

**Keywords:** microglia, evolution, vertebrates, invertebrates, ontogeny, identity, zebrafish

## Abstract

Microglia are unique brain-resident, myeloid cells. They have received growing interest for their implication in an increasing number of neurodevelopmental, acute injury, and neurodegenerative disorders of the central nervous system (CNS). Fate-mapping studies establish microglial ontogeny from the periphery during development, while recent transcriptomic studies highlight microglial identity as distinct from other CNS cells and peripheral myeloid cells. This evidence for a unique microglial ontogeny and identity raises questions regarding their identity and functions across species. This review will examine the available evidence for microglia in invertebrate and vertebrate species to clarify similarities and differences in microglial identity, ontogeny, and physiology across species. This discussion highlights conserved and divergent microglial properties through evolution. Finally, we suggest several interesting research directions from an evolutionary perspective to adequately understand the significance of microglia emergence. A proper appreciation of microglia from this perspective could inform the development of specific therapies geared at targeting microglia in various pathologies.

## Introduction

Microglia were first described as a distinct glial cell type in 1919 by Pío del Río Hortega. They were recognized as very different from the neuroectoderm-derived macroglia, which includes astrocytes and oligodendrocytes (Rio-Hortega, 1920; Penfield, 1932). Studies in mice suggest that microglia originate primarily from the embryonic yolk sac, and this has been confirmed even in humans (Cuadros et al., 1993; Alliot et al., 1999; Rezaie et al., 1999; Chan et al., 2007; Monier et al., 2007; Ginhoux et al., 2010; Schulz et al., 2012; Kierdorf et al., 2013). Yolk sac-derived microglial precursor cells enter the developing mouse brain at around embryonic day (E)8.5 through extravascular routes since the first brain capillaries appear only at E10 (Kurz and Christ, 1998; Navascues et al., 2000; Ginhoux et al., 2010; Swinnen et al., 2013). After taking up residence in the brain, microglia rapidly increase their numbers (Swinnen et al., 2013; Kim et al., 2015; Nikodemova et al., 2015; Eyo et al., 2016). Early entry and subsequent colonization of the brain make microglia well-suited to perform critical roles in regulating crucial events of early brain development, including the phagocytosis-mediated regulation of neural precursor cell numbers, promotion of neuronal cell survival, phagocytosis of dying neurons, synaptic pruning, angiogenesis, axon guidance, synaptogenesis, as well as the refinement and maturation of the developing circuits (Ashwell, 1991; Cuadros et al., 1993; Monier et al., 2007; Wakselman et al., 2008; Fantin et al., 2010; Schafer et al., 2012; Cunningham et al., 2013; Parkhurst et al., 2013; Swinnen et al., 2013; Ueno et al., 2013; Pont-Lezica et al., 2014; Squarzoni et al., 2014).

After a brief period of reduced interest in microglial research following Río Hortega and Penfield, the groundbreaking work of Georg Kreutzberg in 1968 revitalized research interest in microglia by highlighting them as cells responding to facial nerve lesions in mice (Blinzinger and Kreutzberg, 1968). These results were pioneering studies to suggest that microglia can displace synaptic connections following injury. For a long time afterward, microglia in their activated states were identified as neuroinflammatory cells in the context of brain injury, trauma, and disease. Gradually, depending upon the driving stimulus’ context, they were identified with both neuroprotective and neuroinflammatory roles in the brain (Hanisch and Kettenmann, 2007). In maintaining brain homeostasis, microglia constantly survey their external environment continuously sampling neuronal activity through their close physical interactions with neurons, synapses, and other parenchymal brain elements (Davalos et al., 2005; Nimmerjahn et al., 2005; Wake et al., 2009; Tremblay et al., 2010; Schafer et al., 2012; Uweru and Eyo, 2019). They sense their external environment through changes in critical signaling mechanisms that ensure healthy microglia – neuron bidirectional crosstalk that is crucial for maintaining brain homeostasis (Ransohoff and Perry, 2009; Li et al., 2012; Eyo and Dailey, 2013; Eyo and Wu, 2013; Nayak et al., 2014). Subtle changes in this microglia - neuron crosstalk allow microglia to respond to activity-dependent changes, thereby modulating neuronal activity. Microglia remodel synapses in physiology as well as contexts where brain homeostasis is disturbed, such as stress, infection, or injury. Thus, microglia in the mammalian brain serve critical functions in regulating brain homeostasis and immune response to pathology, trauma, or infection (Hong et al.,2016a,b; Fekete et al., 2018; Weinhard et al., 2018). Research over the years has identified microglia to be performing a wide variety of these functions. With the advent of new imaging tools and transgenic models, further insights into the roles of microglia in homeostasis and pathology are being described.

Despite increasing descriptions of microglial roles in physiology and pathology, insight into the evolution of such a specialized cell type is limited primarily because of the lack of comparative microglia studies across different organisms. Among invertebrates, the existence of microglia has been described mainly in the case of phyla Annelida (Rio-Hortega, 1920; Coggeshall and Fawcett, 1964; Morgese et al., 1983; Sonetti et al., 1994), although microglia-like cell types capable of phagocytosis and eliciting an immune response have been described in some other invertebrate such as Drosophila (Leung et al., 2015; Hartenstein and Giangrande, 2018). Therefore, this review highlights some of the important characteristics of microglia that are unique and conserved or divergent across different species with a focus on their molecular identity. We believe that such considerations will facilitate our understanding of the significance of microglia to regulate brain development, facilitate brain homeostatic maintenance, and contribute to brain pathology. In the following pages, we synthesize some of the available evidence for microglia in different species along four lines: their self-renewing capacity, transcriptional identity, developmental ontogeny as well as their roles in physiology and pathology. In doing so, we provide a resource that we hope will generate future research studies in this area.

## Microglia Across Species: A Self-Renewing Brain-Resident Myeloid Population

Microglia have been identified in various invertebrate and all vertebrate species. However, investigations into their ontogeny and maintenance have been especially established in rodents. In the past, there was a serious debate over whether microglia were of neuroectodermal origin as other central nervous system (CNS) cells or of mesodermal origin like other cells of the periphery (Ginhoux and Prinz, 2015). That debate has been largely resolved from studies performed in the mouse. Initial studies revealed that like cells of the myeloid lineage in the periphery, a PU.1 deficiency prevented the development of brain-resident microglia (McKercher et al., 1996; Beers et al., 2006). These findings suggested that microglia and myeloid cells share a similar origin. Although myeloid in origin, the question remained regarding the potential myeloid source(s) of microglia. Resident myeloid cell populations in peripheral organs often consist of circulating bone marrow-derived monocytes that infiltrate into specific tissues and transform into macrophages where they can become tissue-resident. As brain macrophages, it was hypothesized that microglia could originate from ongoing infiltration of circulating monocytes that invade the brain throughout life. Alternatively, a specific source of microglia could exist that seed the brain and maintain microglial populations through cell-autonomous self-renewal.

To begin to address these possibilities, earlier studies used multiple approaches and showed that microglial maintenance does not require infiltration of myeloid cells into the brain in the steady state in mice (Ajami et al., 2007, 2011; Ginhoux et al., 2010). Moreover, more recent studies indicate that when microglia are eliminated from the brain by pharmacological treatment, the subsequent repopulation that ensues does not result from infiltrating myeloid cells but rather from an expansion of the residual brain-resident microglial pool (Huang et al., 2018; Zhan et al., 2019). Moreover, longitudinal visualization of microglia in the adult brain through life repeatedly indicates that individual microglia are long-lived in the mouse (Askew et al., 2017; Fuger et al., 2017; Eyo et al., 2018). Together, these results strongly suggest that microglia are a self-renewing brain-resident population of cells that seed the brain some time in development and are maintained by self-renewal subsequently.

Although the above is true in mice, the extent to which microglial maintenance results from self-renewal rather than peripheral replacement in other species has not been sufficiently explored. It should be noted that at least one study has confirmed that, like mouse microglia, human microglia are long-lived and persist for decades (Reu et al., 2017). Nevertheless, questions of microglial maintenance in the invertebrate leech as well as the vertebrate zebrafish and human need to be further explored.

## Microglia Across Species – Insight From Gene Expression Studies

The advancement of next-generation sequencing techniques over the last decade has facilitated the study of cellular identity and transcriptional expression, especially in the CNS (Dong et al., 2016; Stark et al., 2019). These studies have provided insights into neural cell states and potential functions that can be subsequently validated by other molecular and functional approaches. For microglial identity, these studies over the last 7 years have provided important information on microglial identity at two levels. First, they have clarified a unique microglial transcriptional signature that distinguishes them from other brain-resident cells on the one hand and peripheral myeloid cells on the other. Second, comparative transcriptomics are highlighting conserved and divergent aspects of microglial transcriptional identity across species. In this section, we consider the available data on these two points in turn.

### The Unique Microglial Transcriptional Identity

The question of microglial identity has been clarified in the last decade by the advent of transcriptional profiling. The first microglial transcriptome studies using RNA sequencing compared microglia to other cells of the brain and determined a unique microglial transcriptional signature, including genes such as *P2ry12*, *Tmem119*, and *Hexb* in mice (Hickman et al., 2013; Butovsky et al., 2014; Zhang et al., 2014). Furthermore, when microglia were compared to peripheral macrophages, despite similarities in their myeloid origins and molecular identity, some of the same genes were still uniquely expressed by microglia and lacking in macrophages (Hickman et al., 2013). Further work comparing microglia, monocytes, and perivascular macrophages resident in the brain revealed transcriptional differences between these cells and microglia with the same specific gene set (Goldmann et al., 2016). Therefore, these studies suggest a unique microglial cell signature compared to other brain and immune (including brain-resident immune) cells.

In 2018, to address whether this distinction results from environmental or ontogenic factors, four independent groups arrived at similar conclusions using slightly different approaches. In the first study, using three different approaches to trigger peripheral cells to naturally engraft the adult mouse brain, studies showed that despite long-term residence in the brain, engrafted peripherally derived macrophages fail to adopt the microglial signature including the inability to express microglial specific genes like *P2ry12*, *Tmem119*, and *Sall1* (Cronk et al., 2018). Similarly, a subsequent study using a forced transplantation approach revealed that transplantation of isolated brain-derived microglia fully recapitulated the microglial transcriptional signature. In contrast, transplantation of peripherally derived hematopoietic stem cells (HSCs) failed to do so (Bennett et al., 2018). Engrafted cells also show functionally and transcriptionally distinct responses to stimuli compared to resident microglia (Lund et al., 2018; Shemer et al., 2018). Therefore, these studies indicate that microglia and peripherally derived macrophages are distinct populations in light of their ontogeny though they often exhibit overlapping molecular markers. Together, these studies clarify microglial molecular identity as comprising especially *P2ry12*, *Tmem119*, and *Sall1* among several microglial-specific genes and we use this as the predominant microglial description (It should be noted, though, that one study shows monocyte-derived microglial-like cells with the capacity to express lower levels of *P2ry12* and *Tmem119* but these studies suggest *Sall1* as more defining of microglia).

Despite these findings, more recent studies provide novel insights suggesting that mature microglia are at least partly monocyte-derived. E17 labeled peripheral cells were found in the brain at P2 and P24 expressing microglial-specific *P2ry12*, *Tmem119*, and *Sall1* (Chen et al., 2020). Although the extent to which these monocyte-derived cells contribute to the microglial adult population (i.e., % of total cells and their maintenance into adulthood) is not clear, this finding raises possibilities of converging sources (HSC-dependent and independent sources) of brain microglia in mice (see Section “Microglia across species – insights from microglia in development”). Interestingly, brain transplantation of induced pluripotent stem cells derived from human HSCs into humanized mice showed the ability of these human cells to generate microglia, including the expression of microglial-specific genes (Hasselmann et al., 2019; Svoboda et al., 2019). This suggests that the capacity of HSC-to-microglial transition persists in human cells. Together, these results indicate that endogenous mouse HSCs and exogenous human HSCs retain the capacity to generate microglia during development. Although these results conflict with a previously discussed study during development (Bennett et al., 2018), they may reflect an ancestral capacity for HSC-to-microglial transition that persist in murine and human microglia reminiscent of the situation observed during zebrafish development (Xu et al., 2015; Ferrero et al., 2018). Well-designed studies to ascertain this possibility should, therefore, be conducted.

### Conserved and Divergent Transcriptional Identity Across Species

While the microglial transcriptome has been extensively studied in mice, gene expression studies are beginning to be elucidated in other vertebrates as well. An extensive analysis of the microglial transcriptome across several vertebrate species highlighted the relative homogeneity of microglia among vertebrates. While microglia from various mammalian species like the mouse (including wild mice and four laboratory strains), macaque, marmoset, and hamster showed a single dominant cluster, microglia in humans showed multiple clusters indicating greater heterogeneity (Masuda et al., 2019; Geirsdottir et al., 2020). These results suggest accelerated changes in microglia over the course of human evolution when compared to other non-human vertebrates.

Despite greater microglial diversity in humans than other mammals, microglial transcriptional analysis between vertebrates revealed the conservation of a core set of homeostatic genes. For example, adult zebrafish microglia, like mouse microglia, possess toll-like receptors, chemokine receptors, purinergic receptors, MHC class II, and complement receptor genes among core microglial genes (Oosterhof et al., 2017) suggesting that developmental, homeostatic, and physiological microglial functions regulated by these genes were present in early vertebrates. Similarly, developing zebrafish microglia show a highly conserved transcriptional signature to mouse microglia (Mazzolini et al., 2020). Here, in the latter stages of early development (7 days post-fertilization), microglia exhibit a more diverse clustering than in earlier stages (3 and 5 days post-fertilization), indicating rapid changes in the microglial transcriptome during maturation (Mazzolini et al., 2020). Interestingly, this is reminiscent of the condition in mice where developing microglia are more heterogeneous than adult microglia (Hammond et al., 2019; Li et al., 2019).

In addition to general heterogeneity, microglia in mice show some level of regional diversity. For example, while cerebellar microglia show a more robust phagocytic transcriptional profile, striatal microglia show a weak phagocytic profile in the homeostatic state. These transcriptional observations are consistent with corresponding differences in microglial phagocytic clearance (Ayata et al., 2018). Furthermore, mouse microglia show transcriptional differences by sex (Guneykaya et al., 2018; Villa et al., 2018). However, the extent to which these regional and sex differences in the microglial transcriptome are conserved in lower vertebrates and humans remains to be determined.

In conclusion, assessing the available data for microglial gene expression across species indicates a distinct profile between microglia and peripheral myeloid cells in mice. Although these differences have not been sufficiently explored in other species, microglia in zebrafish and humans show a largely conserved transcriptional profile.

## Microglia Across Species – Insights From Microglia in Development

### Microglial Morphology, Surveillance, and Patrolling Capacities

Microglia exhibit a structure consisting of a central soma and branched processes that emanate from this soma. In all species examined so far, microglia display a ramified structure with their process complexity varying across species. In the invertebrate leech, microglia display a very reduced structural complexity compared to vertebrate microglia (Geirsdottir et al., 2020). However, among vertebrates, while microglia exhibit robust ramifications, each species shows varying degrees of morphological complexity, with marmosets exhibiting the greatest complexity. Humans show an intermediate level of morphological complexity amongst various species examined (Geirsdottir et al., 2020). These results suggest that while there has been a trend toward greater morphological complexity between invertebrate and vertebrate species, vertebrate microglia show highly diverse levels of complexity that is not directly correlated with increasing complexity in phylogenetic evolution. This is unlike the situation with astrocytes that are larger and more complex in humans when compared to mice (Oberheim et al., 2012).

In addition to their complex morphology, microglia are the most morphologically dynamic cells of the vertebrate brain (Davalos et al., 2005; Nimmerjahn, 2012). Microglial basal morphological activity have been characterized with regards to their process dynamics (surveillance) and soma dynamics (migration or patrol). These dynamic features seem to have been well conserved in vertebrates since both zebrafish (Peri and Nusslein-Volhard, 2008; Sieger et al., 2012) and rodent microglia (Petersen and Dailey, 2004; Davalos et al., 2005; Eyo et al., 2016, 2018) exhibit surveillance and patrol. Moreover, chimeric mice with transplanted human microglia also show dynamic process surveillance (Hasselmann et al., 2019).

On microglial patrol, specific developmental features also seem to be conserved across species. For example, in the mouse, microglia display immense migratory capacities in early development in the embryonic cortex (Swinnen et al., 2013) and the early postnatal hippocampus (Eyo et al., 2016). Interestingly, with age, these capacities are rapidly downregulated (Swinnen et al., 2013; Eyo et al., 2016). Similarly, while zebrafish microglia exhibit robust patrol in early development (Peri and Nusslein-Volhard, 2008; Sieger et al., 2012), this patrol is significantly reduced with increasing age during development (Svahn et al., 2013).

Although this progressive reduction in tissue patrol has been documented in mice and fish, neither the reason(s) nor the underlying mechanism(s) for this reduction is clear. One possibility is that intrinsic factor(s) in microglial maturation could account for these changes. Evidence from the zebrafish suggests that between 3 and 5 or 7 days post fertilization (dpf), a time window that corresponds with reduced tissue patrol in zebrafish (Svahn et al., 2013), there is a progressive reduction in migration gene expression (Mazzolini et al., 2020). Conversely, there is an upregulation of *Plxnb2a* in zebrafish (Mazzolini et al., 2020) which in mice negatively regulates macrophage motility (Roney et al., 2011). Therefore, a combination of intrinsic upregulation of migration-inhibition genes and downregulation of migration-promoting genes could be the underlying basis for changes in migration during development. However, this hypothesis remains to be rigorously tested and similar features of the mouse (and human) microglial development need to be carefully explored.

Alternative to intrinsic maturation factor(s) that may regulate microglial patrol in development, extrinsic brain-derived factors could be proposed as instructive cues. One important cue that has been tested is developmental apoptosis. In both mice (Wakselman et al., 2008; Eyo et al., 2016) and zebrafish (Peri and Nusslein-Volhard, 2008; Svahn et al., 2013), microglia aggregate around apoptotic cells in development. Since developmental apoptosis is a transient feature of the developing brain, apoptotic signals could serve as an instructive cue to mobilize microglia. In the fish, independent back-to-back studies showed that apoptosis indeed regulates microglial entry into the brain at a time coincident with high migration (Casano et al., 2016; Xu et al., 2016). However, in mice, while peak microglial patrol occurred with periods of peak apoptosis, elimination of apoptosis did not alter microglial patrol or brain colonization suggesting that apoptosis was not a driving factor in microglial migratory capacity (Peng et al., 2016). Therefore, current evidence indicates that conserved features of microglial dynamic activity during development may be differentially regulated across species.

### The Prevailing Model or Microglial Ontogeny in Mice

In addition to understanding microglial morphological dynamics, studies have been conducted to elucidate microglial ontogeny in development. Microglia were detected in primitive mouse brain tissue as early as E9 (Ginhoux et al., 2010). Myeloid cell populations in the body originate from two broad waves of hematopoiesis termed primitive hematopoiesis (from E7 to E9 in mice) and definitive hematopoiesis (after E9 in mice). To determine which of these sources give rise to brain-resident microglia, Ginhoux et al., 2010 fate-mapped Runx^+^ macrophages at different embryonic ages from E6.5 to E10.5 (Ginhoux et al., 2010). The authors of this study made two notable observations. First, they noted that once labeled as primitive macrophages during these embryonic stages, macrophages persisted into adulthood as microglia at the same ratio as embryonic microglia. Here, E7 labeled macrophages comprised a third of the brain macrophages at both E10 and adulthood, indicating that macrophages that seed the brain in development persist to give rise to adult microglia. Secondly, when primitive macrophages were labeled between E6.5 and E10.5, the percent of adult labeled microglia was low (E6.5 labeling), peaked (E7–E7.5) and then declined (E7.5–E10.5). Together, these results provided powerful evidence that murine microglia seed the brain from embryonically derived yolk sac macrophages that once in the brain give rise to the adult microglial population. Moreover, these studies suggested that later sources of myeloid progenitor cells from the aorto-gonad-mesonephros (AGM) or fetal liver, which are sources of definitive hematopoiesis do not contribute significantly to the adult microglial pool.

Subsequent work has expanded these findings to show that multi-lineage c-kit^+^ erythromyeloid yolk sac precursor cells first originate in blood islands in the embryonic yolk sac at around E7.25 (Ginhoux et al., 2010; Kierdorf et al., 2013). These early yolk sac precursors undergo maturation from A1 (CD45^+^ c-kit^*lo*^ CX3CR1^–^ F4/80^–^) to A2 (CD45^+^ c-kit^–^ CX3CR1^+^ F4/80^*hi*^) amoeboid macrophages that enter and colonize the developing mouse brain at around E8.5 through extravascular routes (Kurz and Christ, 1998; Arnold and Betsholtz, 2013). The first microglia enter the mouse brain through the ventricles or from the meninges by crossing the pial surface and rapidly proliferate and reside in the parenchyma throughout life as brain-resident immune cells (Navascues et al., 2000; Ginhoux et al., 2010; Swinnen et al., 2013; Reemst et al., 2016). They rapidly proliferate between E14 and E16, while slowly peaking until E17.5, and then distribute themselves throughout the brain (Santos et al., 2008; Swinnen et al., 2013).

### Revising the Model for Microglial Ontogeny in Mice

The prevailing hypothesis of microglial origin described above emphasized that adult microglia are *solely* derived from early yolk sac primitive macrophages generated through primitive hematopoiesis without contributions from latter definitive hematopoietic lineage cells that go through the fetal liver (Ginhoux and Prinz, 2015). However, one important feature of the initial study was the fact that at its peak, only a third of the microglial population were labeled in adulthood once fate mapped from the yolk sac in development (Ginhoux et al., 2010). Two explanations could be given for this observation. First, it is possible that the labeling approach inefficiently labels only a third of the primitive hematopoietic macrophage population, which then gives rise to the adult microglial pool. This is the possibility suggested by the Ginhoux et al. (2010). However, an alternative possibility is that adult microglia are composites of both primitive macrophages and alternatively sourced progenitors which would account for the incomplete labeling of adult microglia by primitive macrophages. Interestingly, some recent studies are suggesting alternative aspects of microglial invasion patterns consistent with the second possibility above.

Selective interference with definitive hematopoiesis using *Vav1-Cre^+^:dicer^*fl/fl*^* mice revealed a drastic 40% reduction in microglia at postnatal day (P)1, though not between E16.5-P0 (Fehrenbach et al., 2018) suggesting that a wave of non-yolk sac-derived HSCs contribute to the microglial population through a massive invasion shortly after birth. However, the study also failed to adequately fate map these cells, leaving the possibility that interfering with HSCs may reduce the microglial pool by mechanisms other than direct cell entry. Moreover, whether these “missing” cells are required to constitute the adult microglial pool or whether they are a transient perinatal microglial pool was not addressed. Some of these points were addressed in a different study that labeled liver cells by electroporation at E14 and detected them in the brain at P0 then at P3 when they peaked. However, they were significantly eliminated by P6 (Askew et al., 2017). Therefore, these liver-derived HSCs did not persist as microglia in the mature brain. Whether these are the same cells as those “missing” in the *Vav1-Cre^+^:dicer^*fl/fl*^* mice remains to be determined.

A more definitive fate-mapping study conducted by generating CCR2-Cre^*ER*^ mice has recently been performed (Chen et al., 2020). CCR2 is a monocyte marker that is not expressed by microglia. Chen et al. (2020) labeled CCR2-derivatives at E14 and E17 when definitive hematopoiesis is ongoing. At P2 and also at P24, CCR2-derivatives could be detected in the brain parenchyma, suggesting that these monocytic-derived cells are present in the brain shortly after birth and persist in the brain. Interestingly, these cells take on microglial identity (e.g., expression of *P2ry12, Tmem119*, and *Sall1*, see section “Microglia across species – insight from gene expression studies”) once inside the brain and presumably lose their monocytic identity, which may explain why they had not been detected by previous studies.

In agreement with the foregoing, a different study documents that a specific lineage of adult microglia that express *Hoxb8* transiently in embryonic development when fate-mapped are generated in the yolk sac, then are present in the AGM, then the fetal liver before subsequently taking up residence in the brain (De et al., 2018). In this study, the authors suggested another wave of microglia progenitors that infiltrates the brain at E12.5 and are positive for *Hoxb8*, unlike the microglia progenitor cells seeded at E8.5 (De et al., 2018). But before seeding into the brain, the *Hoxb8* microglia progenitors originating during the second wave of yolk sac hematopoiesis greatly proliferate in the AGM and fetal liver prior to infiltrating the E12.5 brain. Interestingly, the transcriptional analysis indicated that canonical microglia and *Hoxb8*^+^ microglia were highly homogeneous, sharing all but 21 differentially expressed genes. These differences did not affect basal microglial functions in synaptic pruning or chemotaxis (De et al., 2018).

**FIGURE 1:**
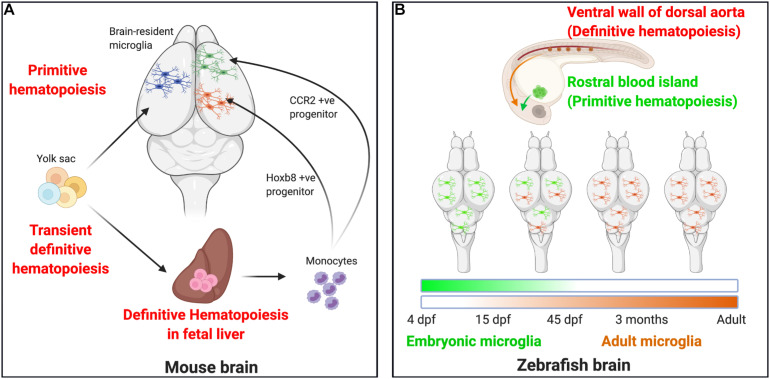
Microglial ontogeny models. **(A)** Mouse: Primitive hematopoiesis in the embryonic yolk sac blood islands at E7.5–E8.0 results in the formation of not just erythrocytes but also highly proliferative macrophage progenitor cells that migrate to the brain. The primitive macrophages colonizing the early developing brain at E8.5 proliferate and give rise to the brain-resident microglia population. A second wave, the transient definitive hematopoiesis in the yolk sac, gives rise to erythromyeloid progenitor cells by E8.0–E8.5 and to T- and B-lymphoid progenitor cells by E9.0. On reaching the fetal liver by E10.5, these cells undergo definitive hematopoiesis to result in the formation of all hematopoietic lineage cells, including monocytes. Recent studies now indicate that definitive hematopoiesis also partly contribute to the brain resident microglia population through hoxb8^+^ and CCR2^+^ monocyte populations taking up residence in the early developing brain parenchyma by E12.5 and E17.0, respectively. **(B)** Zebrafish: Embryonic microglia originating in the rostral blood islands through primitive hematopoiesis by 4 dpf migrates to and populates the embryonic brain until 15 dpf, they disappear entirely by 45 dpf. In essence, the embryonic microglia are replaced by another wave of incoming microglia that arise as a result of definitive hematopoiesis occurring in the ventral walls of the dorsal aorta beginning at 15 dpf. They are the dominant microglia population by 45 dpf and serve as adult microglia throughout the fish’s life.

Despite these independent findings, it is still not clear whether the *Hoxb8*^+^ microglia (De et al., 2018) and the monocyte-turned-microglial cells (Chen et al., 2020) are identical. Nevertheless, these results suggest a necessary revision to the original model of microglial ontogeny in mice such that while the yolk sac seems to be the initial source for brain-resident microglia, two routes are employed for microglial colonization of the brain, i.e., a direct route from the yolk sac to the brain and an indirect route from the yolk sac through hematopoietic intermediates like the AGM and fetal liver before brain arrival (Figure 1A). However, more research is required to make these findings more definitive since for example, the *Hoxb8* line used in these studies is a constitutive line what may alter developmental programming. Moreover, the generation of the CCR2-Cre^*ER*^ mice is new and is yet to be widely validated to confirm.

### An Alternative Model for Microglial Ontogeny in the Zebrafish

Although more work is needed to clarify the picture of microglial ontogeny in the mouse, the evidence provided for microglial ontogeny in the zebrafish is uniform and clearly distinct from that documented in the mouse (Figure 1B). Hematopoiesis in the zebrafish is similar to the mouse in that both primitive and definitive hematopoiesis occur (Davidson and Zon, 2004; Bertrand et al., 2007) (Figure 1B). Primitive hematopoiesis occurs in the rostral blood island (RBI), a region analogous to the mammalian yolk sac, while definitive hematopoiesis occurs in the ventral wall of the dorsal aorta (VDA), a region analogous to the mammalian AGM (Herbomel et al., 1999, 2001; Lieschke et al., 2002; de Jong and Zon, 2005; Bertrand and Traver, 2009; Warga et al., 2009). Microglia are present in the zebrafish brain by 60 h post-fertilization. Using a spatio-temporal light-induced fate-mapping approach, Xu et al. (2015), were first able to show that photolabeling of primitive macrophages in distinct anatomical regions in the zebrafish gave rise to different microglial populations. Early photolabeling of RBI cells gave rise to a high percent of brain-resident microglia at 4 dpf that persisted by 15 dpf but declined by 1.5 months and was virtually eliminated by 3 months. Conversely, photo-labeled VDA cells did not contribute to the microglial population at 4 dpf, began to contribute at 15 dpf, and stably contributed from 1.5 months throughout life (Xu et al., 2015).

Congruent with these observations, another study recently showed that adult microglia from definitive hematopoiesis replace embryonic microglia that originate from primitive hematopoiesis (Ferrero et al., 2018). Interestingly, in the zebrafish, the cMyb transcription factor is required for adult microglial development (Ferrero et al., 2018). However, in the mouse, while cMyb is required for seeding macrophages in other body tissues through the fetal liver, it was not required for seeding microglia in the adult brain (Hoeffel et al., 2015). Nevertheless, given the recent evidence for an indirect colonization route involving transient passage through the liver by a subset of microglia (De et al., 2018), it is possible that at least a subset of mouse microglia indeed require cMyb.

### Interrogating the Model of Microglial Ontogeny in Humans

In humans, amoeboid microglia first penetrate the human cerebral cortex at about 4.5 gestation weeks via the meninges, ventricles, and the choroid plexus. They distribute themselves in the brain through tangential and radial migration (Monier et al., 2007; Verney et al., 2010). For obvious reasons, the ontogeny of human microglia has been poorly interrogated. However, using single-cell transcriptional profiling approaches, a recent study on macrophages from aborted fetuses suggests that human microglia are derived largely from yolk sac progenitors (Bian et al., 2020). Although monocyte-derived cells were detected in the head region, these were only detected at later stages and in smaller proportions (consistent with the revised model in mice), leading the authors to suggest that their contribution to the microglial pool is only slight. Nevertheless, since this study was obtained from only fetal tissues and human studies lack the requisite fate-mapping approach, one cannot rule out a possible expansion of this pool during development as it occurs in mice.

In summary, mouse and zebrafish microglia show salient differences in ontogenic colonization. First, while the zebrafish has a model of replacement of (the equivalent of) yolk sac derived microglia during embryonic development by HSC-derived adult microglia, mouse microglia seem to be derived from the yolk sac but go through at least two routes. Second, the two microglial sources in the zebrafish do not co-exist extensively while they do in the mouse. Thirdly, there may be some molecular distinctions between mouse and zebrafish microglia in mechanisms of brain seeding, especially as relates to the requirement of cMyb. Finally, while incomplete, the picture from human microglial ontogeny suggests features congruent with the situation in mice. Taken from the evolutionary perspective, these differences are significant and would require some explanation that could be better helped by microglial ontogenic studies in other (including invertebrate) species.

## Microglia Across Species: Insights From Described Roles in Physiology

All organisms with a CNS have brain cells of increasing complexity and diversity, including glial cell types that have evolved to take up specialized functions in the brain. In some species, specialized glial cell types have evolved to help with the nourishment and survival of neurons, structural support, removal of metabolic by-products and toxic wastes, and phagocytic needs during pathology or injury conditions. During evolution, microglia emerged as specialized immune cells that function to perceive and respond to pathology and perform a number of homeostatic functions that are essential for the proper functioning of the brain. Although some research has been conducted on microglia in invertebrate mollusks (Peruzzi and Sonetti, 2004; Peruzzi et al., 2004; Sonetti and Peruzzi, 2004), these have been less extensive and we focus our discussion mainly on the invertebrate leech.

### Evidence From Invertebrates (Annelida)

Microglia have been described in annelids in the CNS preparations of the medicinal leech *Hiruda medicinalis* by Pio del Rio-Hortega using silver carbonate staining (Rio-Hortega, 1920). The connective tissue joining neurons of the segmented ganglia of the leech is populated with hundreds of microglia that resemble vertebrate microglia both morphologically in terms of their ramified processes, as well as in terms of showing phagocytic capacity (Coggeshall and Fawcett, 1964; Morgese et al., 1983). However, astrocytes, oligodendrocytes, and infiltrating macrophages are virtually absent from the leech CNS (Le Marrec-Croq et al., 2013). In *in vivo* studies, leech microglia have also been observed to respond to neuronal injury by showing directed migration toward the injury site within minutes, similar to the microglial response seen in vertebrates (Morgese et al., 1983; McGlade-McCulloh et al., 1989). However, unlike microglia in mammals, leech microglia physically translocate their cell bodies in response to injury in minutes while mouse microglia only translocate their processes in the acute response to injury (Davalos et al., 2005; Nimmerjahn et al., 2005).

In the medicinal leech, weak silver carbonate staining can be used to label small amoeboid cells capable of phagocytosis, migration toward the site of injury, expression of nitric oxide synthase (NOS), secretion of antimicrobial peptides in response to infection, as well as in expression of purinergic receptors that induce migration in response to adenosine triphosphate (ATP) (Morgese et al., 1983; Shafer et al., 1998; Schikorski et al., 2008; Duan et al., 2009). In vertebrates, microglia/macrophages during an immunological response upregulate the expression of ionized calcium-binding adapter molecule 1 (Iba1), also known as allograft inflammatory factor 1 (Aif-1), which also serves as a reliable marker to label these cells (Ito et al., 1998; Beschorner et al., 2000; Schwab et al., 2001). Similarly, Drago et al. (2014) first reported the expression of an invertebrate counterpart of Iba1, *Hm*Iba1, in leech microglia that were seen migrating and accumulating at the lesion site upon injury or ATP stimulation. However, *Hm*Iba1 expression was very low in naïve or unstimulated conditions but rapidly increased following an immunological insult (Drago et al., 2014). Therefore, most of the strong immunoreactivity toward *Hm*Iba1 was localized to microglia migrating or accumulating at the injury or stimulation site in the leech CNS preparations.

The migration and clustering phenotype of leech microglia is especially evident in experimentally induced nerve lesion studies in the segmental ganglia, where microglia migrate to and cluster at the injury site to phagocytose and remove dead neurons (Morgese et al., 1983). This not only facilitates clearance of the debris from the injury site but also aids in the initiation or completion of any subsequent repair mechanisms. Microglial chemotaxis to the injury site is mediated by nitric oxide (NO) signaling, as is evident by the increased NOS immunoreactivity at the injury site at 24 h corresponding to the peak in microglia numbers at that time (Chen et al., 2000). This is further supported because prior inhibition of NO synthesis using NOS inhibitor L-NAME effectively prevents microglial chemotaxis response and clustering at the injury site. Similarly, increasing NO levels using spermine NOate (SPNO), a NO donor, also inhibits microglia accumulation, possibly due to reduced chemotaxis resulting from the loss of NO gradient and saturation in the microglial microenvironment (Chen et al., 2000).

The leech microglia resemble vertebrate microglia in their capacity of demonstrating chemotaxis and response to injury and clearing of the dead neurons by phagocytosis to aid in the initiation of neuronal repair mechanisms. Experiments using isolated leech nerve cord preparations of leech have also demonstrated the basal process movement and chemotaxis of leech microglia to ATP gradients, much like that seen in vertebrates. Based on the experimental evidence, basal level movement of leech microglial cells is thought to be regulated by levels of ATP, ADP, and UTP, and several candidate receptors, including P2RY2- or P2RY4-like receptors, are thought to be involved in mediating this response (Duan et al., 2009). Using the nerve crush model in the isolated nerve cord preparations, leech microglia were also demonstrated to show chemotaxis to ATP application at the injury site and is mediated through P2 receptors since its pharmacological inhibition using reactive blue 2, a non-selective antagonist for P2Y, resulted in a decline in microglia accumulation at the injury site (Duan et al., 2009).

Just like the mammalian proinflammatory cytokine IL-16, leech microglia show chemotactic activity toward *Hm*IL-16, a cytokine possibly first released by damaged neurons leading to microglial activation and subsequent release of *Hm*IL-16 molecules to recruit more microglia to the site of injury (Croq et al., 2010). Furthermore, in an *in vitro* chemotaxis assay, anti-human IL-16 antibody or anti-*Hm*IL-16 antibody preincubation of microglial cells led to a significant decline in microglia migration induced by CNS-conditioned medium collected from injured leech (Le Marrec-Croq et al., 2013).

*Hm*C1q is another key molecule critical for microglial functions in the leech, just as its vertebrate counterpart C1q is important for microglial activity in vertebrates (Tahtouh et al., 2012). Leech neurons and glial cells release *Hm*C1q that serves as a dose-dependent chemoattractant for microglia to aid in further microglia recruitment and is sensitive to treatment with anti-*Hm*C1q antibodies. Also, microglia at the nerve injury site in leech strongly express the receptor *Hm*gC1qR or *Hm*C1qBP for *Hm*C1q, and treatment with anti- *Hm*C1qBP antibodies leads to the inhibition of *Hm*C1q-mediated microglial migration in *in vitro* preparations (Tahtouh et al., 2012). Leech *Hm*C1q can also interact with calreticulin (*Hm*CalR), expressed by a small population of microglial cells at the injury site. This interaction can result in *Hm*C1q-dependent microglia accumulation (Le Marrec-Croq et al., 2014).

In summary, several features of vertebrate microglia, including Iba1 protein expression, purinergic, cytokine- and NO-dependent responses, seem to be conserved in the invertebrate leech as in vertebrates indicating that core features of microglial identity and function were present early in microglia during evolution and have been preserved through evolution. Because the medicinal leech has not been extensively investigated, future work will be needed to clarify the specifics and extent of divergence in microglial function between invertebrates and vertebrates. Therefore, there is a need to develop of tools to effectively study microglia in invertebrates as has been done for vertebrate species.

### Evidence From Vertebrates

Microglia are specialized neuroimmune cells that exhibit neuroprotective traits throughout development and adulthood. Abnormal and dysregulated microglia could play a part in the occurrence and maintenance of neurological and degenerative disorders and psychiatric illnesses due to their closeness to other cellular elements of the brain, including neurons. The neuroprotective invertebrate microglia seem to have evolved amplified neuroprotective capabilities to participate in different roles in vertebrates, including crucial roles in brain development and maintenance of brain homeostasis. Within this context, vertebrate microglia offer model systems to investigate underlying cellular and molecular mechanisms involved in the maintenance of higher-order neuroimmune functions by mammalian microglia. Below we consider a few of these established functions of microglia.

#### Apoptosis and Brain Development

Microglia actively eliminate excess neurons during early brain development by inducing apoptosis, or they may respond to the ongoing neuronal cell death by phagocytosis. In work done by Lang and Bishop (1993) on the role of macrophages in the mouse developing eye, Diphtheria toxin-mediated ablation of microglia resulted in the persistence of two ocular tissues, namely, the hyaloid vasculature and the pupillary membrane. During normal eye development, these issues are typically removed by macrophage-mediated apoptosis, thus providing evidence for the role of macrophages in actively inducing apoptosis of excessive cells before phagocytosing them. Wakselman et al. (2008) also demonstrated superoxide-induced neuronal apoptosis in the hippocampus by microglia as a result of cluster of differentiation molecule 11b (CD11b) and DNAX-activating protein of 12 kDa (DAP12) activity. They further observed a decline in neuronal apoptosis in the developing hippocampus in both transgenic mice deficient for microglial proteins CD11b or DAP12 or in wild-type mice treated with function-blocking antibodies. Furthermore, in the mouse cerebellum, microglia have also been implicated in the induction of apoptosis of developing purkinje cells via superoxide ion release (Marin-Teva et al., 2004).

As in the mouse, microglia display phagocytic activities in the developing chick retina (Frade and Barde, 1998) and macaque brain (Cunningham et al., 2013). In the zebrafish, microglia are also highly phagocytic in development. Indeed, available evidence suggests that they may be more efficient at phagocytosis than microglia in mice since virtually all apoptotic debris can be found within microglia in the normal zebrafish during development (Peri and Nusslein-Volhard, 2008; Svahn et al., 2013) unlike in mice where some apoptotic debris remains unengulfed by microglia at any given moment (Wakselman et al., 2008; Eyo et al., 2016). These findings may suggest downregulation of phagocytic clearance efficiency across evolution, requiring more careful characterization of phagocytic mechanisms across species. However, the presumed efficiency in phagocytosis between zebrafish and mouse microglia might result from the amount of available debris, i.e., mouse microglia may be “less efficient” at clearance because there is more debris in the mouse brain than in the zebrafish brain during development. Indeed, in the neurogenic niche where apoptosis is not overwhelming, mouse microglia are more efficient at clearance (Sierra et al., 2010; Abiega et al., 2016).

During brain development, microglia have also been observed to promote neuronal cell survival by the secretion of supporting neurotrophic factors. Ueno et al. (2013) found that inhibiting microglial activity resulted in an increase in neuronal apoptosis in layer V of the postnatal day 3 – 5 aged mouse cerebral cortex in minocycline treated mice. Primary cultures prepared from embryonic E12/E13 Pu.1 knockout mice brains show a decreased proliferation of neural precursor cells (Antony et al., 2011). Furthermore, microglia are critical in circuit wiring of the developing mouse brain (Squarzoni et al., 2014; Zhan et al., 2014). The extent to which these features of microglial function are present in zebrafish, chick, macaques, and humans remains to the determined and should be an interesting line of future work.

In addition, microglia are also instrumental in vascular development and maintenance of vascular integrity following injury. In mouse embryonic development, microglia were shown to associate with the vasculature and suggested to promote the connection of blood vessel tips, otherwise known as anastomosis (Fantin et al., 2010). This was deduced in the mouse embryo by analyzing fixed embryonic tissue or imaging in *ex vivo* slices, leaving the possibility that such actions were an artifact. However, the authors further confirmed these observations in the zebrafish with the power of live imaging of the developing organism (Fantin et al., 2010). Similarly, in both the mouse (Lou et al., 2016) and the zebrafish (Liu et al., 2016), microglia were shown to repair the injured vasculature. In both species, the purinergic P2RY12 receptor was required for microglial process (mouse) or cell body (zebrafish) migration to the injured vessel, suggesting vascular complexity development, vascular targeting mechanisms, and vascular repair functions are conserved between fish and mice.

#### Synapse Refinement and Formation

In the postnatal brain, microglia also function to refine excessive synaptic connections formed as a result of synaptogenesis via synaptic pruning mechanisms that involve the selective elimination of synapses by phagocytosis of pre- or post-synaptic elements (Chechik et al., 1998; Kano and Hashimoto, 2009; Schafer and Stevens, 2013). Microglia and astrocyte-mediated synaptic pruning was first observed during local neural circuit refinement in the developing cat brain corpus callosum (Berbel and Innocenti, 1988). Similarly, in the P5 retinogeniculate system, C3 receptor-expressing microglia prune out synapses expressing high levels of C3 protein via a complement-dependent phagocytosis mechanism. Disruption of this synaptic pruning of retinal axons (as in mice lacking the C3 complement or its receptor) showed a sustained deficit in eye-specific segregation, thus stressing the crucial synaptic pruning roles of microglia in sculpting neural circuitry and refining the neuronal landscape in the early postnatal brain (Gasque, 2004; Stevens et al., 2007; Schafer et al., 2012). While remodeling and refining neuronal circuitry in the brain, microglia also use their ramified processes to perform synaptic stripping function that involves the physical separation of pre- and post-synaptic elements (Blinzinger and Kreutzberg, 1968; Tremblay and Majewska, 2011). While synaptic pruning by microglia has been extensively investigated in the mouse, this work has not been explored in other species, including the zebrafish.

In addition to their pruning activity, microglia can also induce new filopodia extension that may stabilize to form new synapses. Miyamoto et al. demonstrated microglia-induced new filopodia extension in layer 2/3 pyramidal neurons of the developing mouse somatosensory cortex due to increased Ca^2+^ transients and actin accumulation at the microglia contact sites on the dendrite (Miyamoto et al., 2016). Furthermore, minocycline-induced microglia inhibition or pharmacogenetic ablation of microglia resulted in a marked decline in dendritic spine density and functional excitatory synapse numbers (Miyamoto et al., 2016). In the adult mouse brain, synapse formation following a motor learning task was also promoted by microglia (Parkhurst et al., 2013). Nevertheless, similar to synaptic pruning, microglial regulation of synapse formation has not been explored in other species. Therefore, the extent to which these notable functional microglial activities are conserved across vertebrates cannot be currently ascertained.

## Conclusion and Outstanding Questions

Microglia play critical roles in the brain, serving as the first responders to trauma or pathogens in invertebrates and vertebrates. Based on the limited invertebrate microglia studies available, microglial functions in invertebrates are largely restricted to responding to pathogenic invasion, neural protection, and repair in injury. However, microglia have been shown to play additional crucial roles in the wiring of the vertebrate CNS and the maintenance of brain homeostasis. With the increasing complexity of the CNS through evolution both in terms of structural organization and functions, glial cells arose in response to the need for specialized supporting cells to assist neurons in their survival in conditions of injury or pathological invasion (Hartline, 2011). Specialized immune cells of mesodermal origin like professional macrophages are known to circulate in the body cavity of the Drosophila, where they are often found closely adjacent to sensory neurons in the body cavity. Based on this observation, Hartenstein and Giangrande (2018) raised the possibility of invading immune system cells taking up residence in the CNS to display microglia-like functions such as phagocytosis. The need for additional mechanisms mediating the entry of macrophage progenitors into the CNS to become microglia suggests an important evolutionary advantage that microglia provide to the vertebrate CNS. With growing interest in microglia during development and neurodegeneration, a better understanding of microglial biology as it has developed through evolution is important.

Examining microglia from an evolutionary perspective is in its infancy but our current summary of salient aspects of comparative microglial biology across species is essential to such an evolutionary understanding. To conclude this review, we discuss some of the outstanding questions that need to be tackled to move the field forward. Much work needs to be done to understand microglial identity, ontogeny and physiology across different species. These questions have only been studied so far in a limited number of model organisms. Although microglia have been identified in both vertebrates and invertebrates (phyla Annelida), it is not clear that these are identical cells since they are not present in all shared phyla (e.g., Arthropoda, which includes *Drosophila*) that emerged from the common ancestral groups that include both annelids and vertebrates. This suggests that it is likely that these cells emerged independently (Hartenstein and Giangrande, 2018). If so, similarities and differences between invertebrate and vertebrate microglia would be of interest to indicate added, lost or independently converging functions across evolution.

**FIGURE 2:**
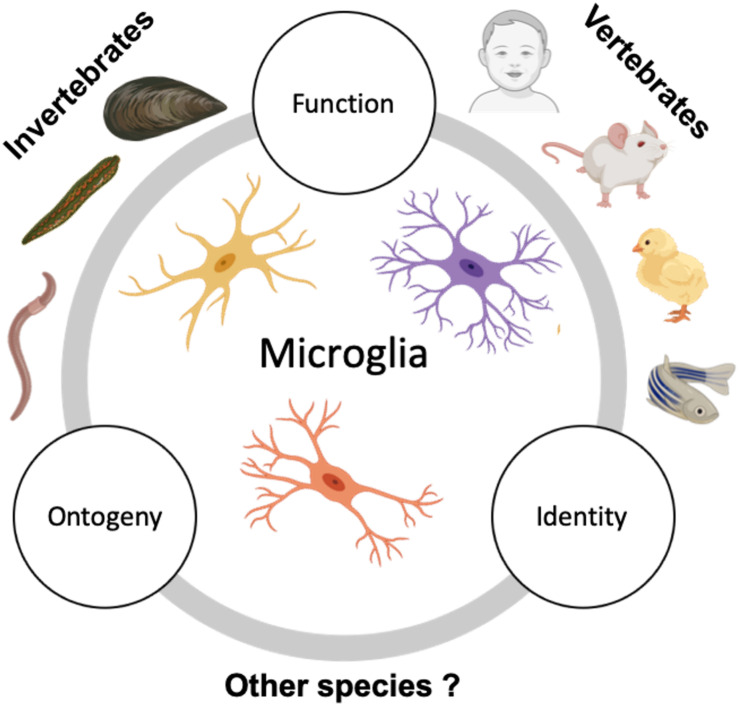
Suggested future comparative studies on microglia across species. Studies describing microglia in invertebrates are sparse and usually focus on microglia seen in phyla Annelida (in leeches and earthworms) and Mollusca (in mussels). However, the ontogeny and detailed characterization of invertebrate microglia are still lacking. Contrary to this, vertebrate microglia, especially zebrafish and mammalian microglia have been extensively studied and characterized for their origin, identity, and function. Additional study of microglia in the existing invertebrate models in conjunction with expanding the search to other invertebrate species and examining them with respect to their ontogeny, identity, and function in relation to vertebrate microglia could help us gain insight on the significance of microglial emergence in evolution.

The leech remains the most extensively studied invertebrate species for microglia, making our knowledge of invertebrate microglia severely limited. Explorations into other invertebrate microglia will thus provide a better understanding of invertebrate-evolved microglia. Among vertebrates, our understanding of microglial cell functions is largely derived from studies in mice and growing investigations in the zebrafish. While there were some developmental microglial studies in the chick at the end of the last and the beginning of this century, chick models to interrogate microglia have lost favor with the wealth of tools available for mice and zebrafish as model systems. However, comparative (i) identity, (ii) ontogenic, and (ii) functional studies across vertebrate species are of pressing need. Several questions remain along these three lines (Figure 2).

### Identity

First, the question of microglia identity across species remains to be comprehensively tackled. Since microglial identity has now been clarified at the molecular level (*P2ry12*, *Tmem119*, and *Sall1* along with several microglial-specific genes), they can be identified across species and adequately compared. Microglial heterogeneity is now well-documented in mice at the molecular level (Chen et al., 2010; Ayata et al., 2018; Kana et al., 2019), between development and adulthood (Hammond et al., 2019; Li et al., 2019) as well as on the basis of sex (Guneykaya et al., 2018; Hanamsagar et al., 2018). However, this heterogeneity pales when compared to the heterogeneity in humans (Geirsdottir et al., 2020). Furthermore, microglial heterogeneity is recognized in zebrafish (Wu et al., 2020). However, the extent to which these heterogenous microglial populations are already present in ancestral species remains to be adequately clarified. Moreover, certain classical molecular identifiers may not be present in all microglial species e.g., Iba1 (which labels brain macrophages and microglia) fails to label fish or chick microglia (Geirsdottir et al., 2020) though it labels leech microglia (Drago et al., 2014). Does this render the microglia from these species differentially functional from others, and is this feature (lack of Iba1 expression) specific to these species (zebrafish and chick) or are they features of other microglia from other species? Moreover, there is growing evidence of sexually dimorphic transcriptional and translational expression in mouse microglia (Guneykaya et al., 2018; Hanamsagar et al., 2018; Villa et al., 2018). To what extent this is conserved across both vertebrate and invertebrate species remains to be determined. With the currently available tools and background knowledge provided above, these questions can now begin to be addressed.

### Ontogeny

Thus far, species-specific microglial ontogenic studies have been conducted in the mouse and zebrafish. Results with cell fate-mapping approaches indicate distinct mechanisms employed in these species in brain colonization during development. For example, while mouse microglia are derived from yolk sac macrophages, seed the brain and persist therein through life, zebrafish microglia have distinct waves of embryonic and adult microglia in which the latter replace the former. These are fundamentally distinct ontogenic mechanisms. Whether these mechanisms are uniquely employed by each of these species and how these mechanisms evolved will have to be a focus of future research. Moreover, while it is clear that mouse microglia are a self-renewing population in adulthood, i.e., lacking homeostatic contributions from peripheral cells, it is not clear whether this is the case for microglia from non-mammalian vertebrate (or invertebrate) species. Therefore, clarifying microglial ontogenic and homeostatic maintenance mechanisms across vertebrate (and invertebrate) species should be a subject of future research.

### Functional

As a third and final area of research interest, cross-species functional microglial studies need to be conducted. The majority of our understanding of microglial functions in health and pathology comes from rodent systems with additional insights from zebrafish and humans. While certain features of microglial physiology, e.g., phagocytic clearance and chemotactic response to purines have been confirmed in vertebrates and invertebrates, (a) comparative abilities between species remain to be determined, and (b) the extent of salient rodent microglia functions remains to be confirmed in other species including, e.g., synapse elimination/formation (Parkhurst et al., 2013; Miyamoto et al., 2016) or memory/forgetting (Wang et al., 2020). While we now know many of the roles employed by microglia in the mouse such as recent evidence for microglial maintenance of myelin (Olah et al., 2012; Hagemeyer et al., 2017), it remains unclear whether these roles have been employed by microglia since their emergence or whether they are newly added roles. Are microglia in the fish or the leech equally capable of the functions attributed to them in the mouse? This would be an interesting line of research focus for the future.

In summary, we have evaluated microglia from a comparative species perspective highlighting several insights obtained from primarily rodent models on their maintenance, identity, ontogeny, and function, while comparing this knowledge to the little we know on these points from other species. Our assessment suggests there are intriguing unanswered questions from this perspective, which we have highlighted and recommend for future studies. We trust that with this knowledge, we will have a better understanding of the significance of microglial emergence in evolution.

## Author Contributions

All authors listed have made a substantial, direct and intellectual contribution to the work, and approved it for publication.

## Conflict of Interest

The authors declare that the research was conducted in the absence of any commercial or financial relationships that could be construed as a potential conflict of interest.

## Abbreviations

CNS: central nervous system
dpf: days post-fertilization
E(): embryonic day ()
AGM: aorto-gonad-mesonephros
CD: cluster of differentiation
P(): postnatal day ()
HSC: hematopoietic stem cells
RBI: rostral blood island
Iba1: ionized calcium-binding adapter molecule 1
NO: nitric oxide
NOS: nitric oxide synthase
ATP: adenosine triphosphate
SPNO: spermine NOate
IL: interleukin
CD11b: cluster of differentiation 11b protein
DAP12: DNAX-activating protein of 12 kDa
BDNF: brain-derived neurotrophic factor
IGF1: insulin-like growth factor 1.
